# Rezidiv einer septischen Arthritis des Handgelenks durch *Pseudomonas aeruginosa* bei Besiedelung eines zentralvenösen Verweilkatheters

**DOI:** 10.1007/s00393-020-00842-y

**Published:** 2020-07-23

**Authors:** C. Thibeault, U. Schneider, A. Eisenschenk, M. Lautenbach

**Affiliations:** 1 Medizinische Klinik mit Schwerpunkt Infektiologie und Pneumologie, Charité – Universitätsmedizin Berlin, Campus Virchow Klinikum, Augustenburger Platz 1, 13353 Berlin, Deutschland; 2 Medizinische Klinik mit Schwerpunkt Rheumatologie und Klinische Immunologie, Charité – Universitätsmedizin Berlin, Campus Mitte, Berlin, Deutschland; 3 Hand- und funktionelle Mikrochirurgie, Universitätsmedizin Greifswald, Greifswald, Deutschland; 4 Abteilung Handchirurgie, obere Extremität und Fußchirurgie, Krankenhaus Waldfriede Berlin, Berlin, Deutschland

**Keywords:** Septische Arthritis, Infektiöse Arthritis, Bakterielle Arthritis, Monoarthritis, Biofilm, Septic arthritis, Infectious arthritis, Bacterial arthritis, Monoarthritis, Biofilm

## Abstract

Ein Diabetiker, Träger eines Ports und mit Zustand nach Gonarthritis durch *Pseudomonas aeruginosa*, erlitt eine subakute Arthritis eines Handgelenks. Protrahiert gelang der kulturelle Nachweis von *P. aeruginosa* aus dem explantierten Port und dem betroffenen Gelenk. Der Fall zeigt, dass bei Patienten mit unklarer Handgelenkarthritis, Vorgeschichte einer septischen Arthritis mit *P. aeruginosa* und Risikofaktoren für eine hämatogene Streuung ein Rezidiv ausgeschlossen werden sollte. Die Therapie bestand aus Portexplantation, Débridement mit Synovialektomie des Gelenks und antibiotischer Therapie.

## Abstract (en)

A diabetic patient, wearer of a port and with a history of gonarthritis due to *Pseudomonas aeruginosa* presented with subacute arthritis of a wrist. After a protracted interval *P. aeruginosa* was detected by microbial culture from the explanted port and the affected joint. This case shows that in patients with unclear arthritis of the wrist, a history of septic arthritis with *P. aeruginosa* and risk factors for hematogenous spread, a recurrent infection should be excluded. The treatment consisted of explantation of the port, débridement with synovectomy of the joint and adequate antibiotic treatment.

## Falldarstellung

### Anamnese

Ein 65-jähriger Taxifahrer wurde stationär eingewiesen mit einer seit 7 Wochen bestehenden progredient schmerzhaften Schwellung und Bewegungseinschränkung des rechten Handgelenks ohne erinnerliches Trauma oder äußere Verletzung. Der Schmerz erreichte morgens und bei Bewegung ein Maximum nach visueller Analogskala 10/10.

Anamnestisch waren ein Nikotinkonsum, ein nicht insulinpflichtiger Diabetes mellitus Typ 2 sowie eine vorausgegangene Podagra bekannt. Vier Jahre vor der aktuellen stationären Aufnahme war ein Rektumkarzinom operiert und mit Radiochemotherapie adjuvant behandelt worden. Der in diesem Rahmen implantierte zentralvenöse Verweilkatheter (Port) war bisher nicht explantiert worden, der Patient war bis dato tumorrezidivfrei. Zwei Jahre vor der aktuellen Aufnahme und anderthalb Jahre nach Abschluss der Chemotherapie hatte der Patient eine Gonarthritis mit Kniegelenkempyem rechts mit intraartikulärem Nachweis von *P. aeruginosa* erlitten. Ein Fokus war nicht eruierbar. Die Behandlung war arthroskopisch mit Spülung und Synovialektomie sowie antibiotisch erfolgt. Bei Aufnahme war der Patient nach erfolgter Punctio sicca des betroffenen Handgelenks bereits auswärtig ambulant unter dem Verdacht einer Arthritis urica mit Prednisolon 50 mg/Tag sowie Diclofenac und Metamizol anbehandelt worden, worunter keine Besserung erzielt wurde. Er nahm außerdem dauerhaft Metformin, ASS, ein Statin und einen Betablocker ein.

### Befund

#### Klinische Untersuchung

**Figure 1:**
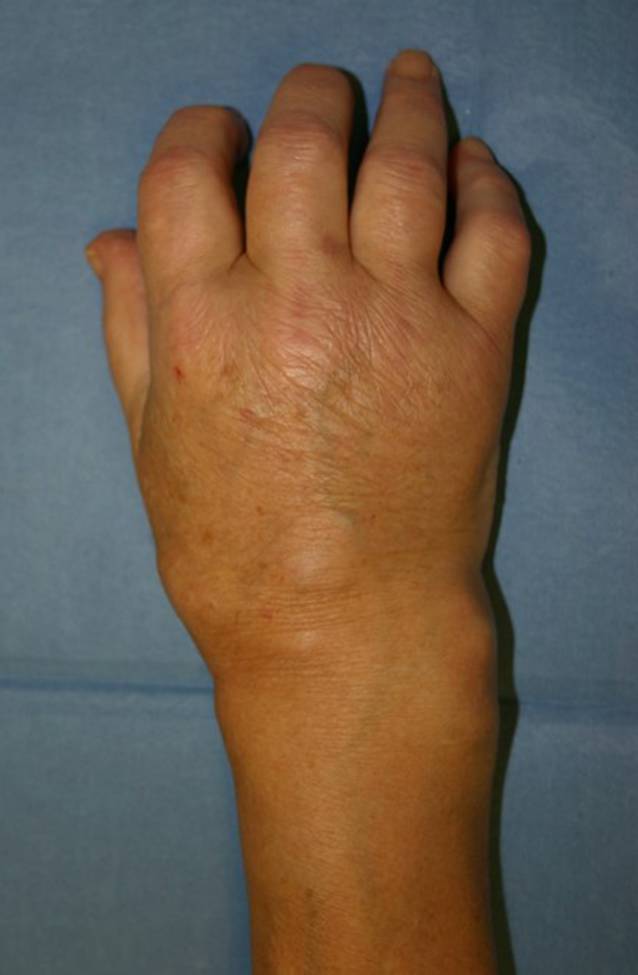

Bei der körperlichen Untersuchung fiel ein schmerzhaftes, gerötetes, überwärmtes und geschwollenes rechtes Handgelenk mit Ausbreitung der Schwellung über den Handrücken auf (Abb. 1). Die Bewegungsmaße im Handgelenk waren schmerzbedingt in Extension und Flexion eingeschränkt (nach Neutral-Null-Methode 20/0/20), der Faustschluss aller Finger inkomplett. Der Daumen war schmerzbedingt endgradig bewegungseingeschränkt. Der Patient war fieberfrei. Der Narbenbereich des einliegenden venösen Ports war klinisch unauffällig.

#### Sonographie und Punktat des Ergusses

Sonographisch zeigte sich ein minimaler Erguss des betroffenen Handgelenks. Das Punktat war leukozytenreich und mikroskopisch frei von Kristallen. Ein Erregernachweis aus dem Punktat gelang nicht.

#### Radiologie

**Figure 2:**
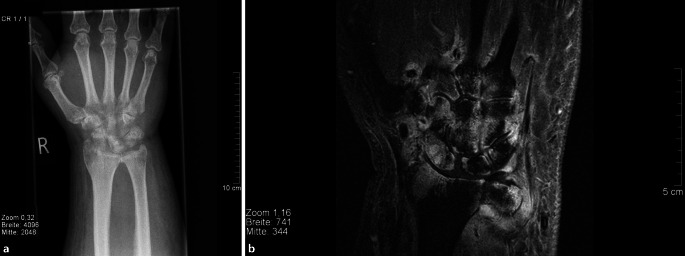

Im Röntgenbild des Handgelenks in 2 Ebenen und der Magnetresonanztomographie zeigten sich eine Osteitis des distalen Unterarms und gesamten Carpus mit deutlichen Erosionen sowie eine Handgelenksynovialitis. Zusätzlich bestand eine Synovialitis im Bereich der Strecksehnenfächer (Abb. 2).

#### Labor und Mikrobiologie

Die Entzündungsparameter waren unter Einnahme von Prednisolon leicht erhöht: BSG 18 mm/h, CRP 5,8 mg/l (Referenzbereich bis 5 mg/l), Leukozyten 12,10/nl (Referenzbereich 3,9–10,50/nl).

#### Weitere Diagnostik

Die infektiologische Umgebungsdiagnostik auf auslösende Erreger einer reaktiven Arthritis (Yersinien, Chlamydien) blieb ebenso negativ wie die Lues- und Borrelienserologie und ein Urethralabstrich auf Gonokokken. Die Bestimmung von HLA-B27, Rheumafaktoren und rheumatologischen Antikörpern war negativ.

## Diagnose

Im Verlauf des stationären Aufenthaltes entwickelte der Patient eine venöse Thrombose der V. jugularis interna, subclavia und axillaris dextra, weshalb der venöse Port explantiert wurde. In den aus dem Portabstrich entnommenen Kulturen zeigte sich ein Wachstum eines multisensiblen *P. aeruginosa*, sodass bei fehlendem Wachstum in den peripher abgenommen Blutkulturen von einer Besiedelung des venösen Portsystems ausgegangen wurde und eine bakterielle septische Arthritis (SA) vermutet wurde.

## Therapie und Verlauf

Es erfolgte die Synovialektomie des Handgelenks und der Strecksehnenfächer mit gründlichem Débridement, Wundspülung und Drainageneinlage. Auch intraoperativ konnte ein multisensibler *P. aeruginosa* nachgewiesen werden. Im weiteren Verlauf wurden eine Wundrevision und ein Débridement der infizierten Porttasche durchgeführt. Beide Operationen verliefen komplikationslos. Postoperativ erfolgte die kalkulierte antibiotische Therapie mit Cefuroxim und Ciprofloxacin i.v., die nach 3 Tagen resistenzgerecht auf eine Monotherapie mit Ciprofloxacin umgestellt wurde (2-mal täglich 750 mg über 7 Tage i.v. und Fortführung über weitere 14 Tage mit 2‑mal täglich 500 mg p.o.).

Bei Entlassung aus der stationären Therapie (15 Tage nach der ersten Operation) bestanden reizfreie Wundverhältnisse und Schmerzfreiheit der gesamten Hand. Nach intensiver Hand- und Physiotherapie gelang eine deutliche Besserung der Funktionseinschränkung. Das Handgelenk zeigte ein Bewegungsausmaß von ROM 85° in Streckung und Beugung (Extension/Flexion 45-0-40) bei endgradig eingeschränkter Radial- und Ulnarduktion. Der Faustschluss aller Finger sowie die Daumenbewegung waren frei. Ein Kompressionshandschuh nach Maß wurde für 3 Monate getragen.

## Diskussion

Wir beschreiben hier den Fall eines Rezidivs einer SA mit *P. aeruginosa* 2 Jahre nach der ersten Episode an unterschiedlicher Lokalisation. In dem beschriebenen Fall war insbesondere die differenzialdiagnostische Abgrenzung zur Arthritis urica schwierig, da anamnestisch und klinisch für beides Hinweise vorlagen. Beide Krankheitsbilder können zudem simultan vorliegen, und ein fehlender Erregernachweis schließt die septische Arthritis nicht aus, wie auch ein fehlender Kristallnachweis die Arthritis urica nicht ausschließt. In unserem Fall machte die fehlende Besserung auf eine Glukokortikoidtherapie die Arthritis urica unwahrscheinlich. Die Entzündungsparameter waren unter Glukokortikoidtherapie nur eingeschränkt verwertbar. Letztlich war der radiologische Nachweis destruierender Veränderungen 10 Tage nach Symptombeginn wegweisend für eine infektiöse Genese. Die Ätiologie der ersten SA lässt sich nicht mehr klären. Unsere Hypothese bezüglich des hier beschriebenen Rezidivs ist, dass es vor oder im Rahmen der ersten Episode zu einer anhaltenden Besiedelung des venösen Portsystems mit *P. aeruginosa* kam, die trotz adäquater antibiotischer Therapie nicht eradiziert wurde und ursächlich für die hämatogene Streuung und damit die hier beschriebene Episode der SA des rechten Handgelenks war. Die Symptomfreiheit unseres Patienten zwischen erster SA und Rezidiv lässt vermuten, dass sich auf dem venösen Portsystem ein Biofilm mit *P. aeruginosa* gebildet hat. Elastische Biofilme sind oberflächenassoziierte Bakterienkolonien umgeben von extrazellulärer Matrix und stellen eine gut beschriebene bakterielle Überlebensstrategie dar, die diese vor Antibiotika und Immunantwort des Wirts schützt [2]. Sie bieten ein Reservoir für katheterassoziierte Infektionen [6] und sind eine mögliche Erklärung für die zunehmende Inzidenz medizinproduktassoziierter septischer Arthritiden (SA) [9]. Zu vermuten ist auch, dass die bei vorbestehender Tumorerkrankung mit Chemotherapie bestehende Immunsuppression sowie der vorliegende DM Typ 2 des Patienten eine Biofilmformation begünstigt und/oder das Auflösen derselben verhindert haben. Im Tiermodell können immunsupprimierte und diabetische Tiere *Pseudomonas-aeruginosa*-Biofilme schlechter beseitigen als gesunde [13].

Unserem Kenntnisstand nach gibt es keine evidenzbasierten Empfehlungen bezüglich des Umgangs mit langfristigen venösen Verweilkathetern bei Patienten mit SA ohne Hinweis für eine katheterassoziierte Infektion. Der hier beschriebene Fall legt jedoch nahe, dass durch eine frühzeitigere Entfernung des venösen Portsystems das Rezidiv hätte vermieden werden können.

Die SA geht, abhängig von ambulantem oder nosokomialem Erwerb der Infektion, Alter und betroffenem Gelenk mit einer 30-Tages-Mortalität von bis zu 26 % und einem Funktionsverlust des Gelenks in bis zu 50 % einher [4, 10]. Eine unverzügliche Diagnostik und Therapie ist prognoseentscheidend. Die Infektion des Gelenks wird entweder durch hämatogene Streuung, direkte Inokulation im Rahmen eines Traumas mit lokaler oder regionaler Fortleitung oder iatrogen hervorgerufen. Generelle Risikofaktoren für die SA sind Alter >80 Jahre, Diabetes mellitus, HIV, rheumatoide Arthritis, bestehende Gelenkendoprothesen oder vorausgegangene Gelenkoperationen und Hautinfektionen [3, 7]. Ein erhöhtes Risiko für eine hämatogene Ursache einer septischen Arthritis haben immunsupprimierte und hospitalisierte Patienten, insbesondere wenn bei diesen Gefäßkatheter oder größere invasive Eingriffe notwendig waren [8].

Das typische klinische Bild der septischen Arthritis ist eine akute Monoarthritis. Häufige Differenzialdiagnosen der akuten Monoarthritis sind neben der Infektion durch Bakterien, Pilze oder Mykobakterien unter anderem reaktive Arthritiden, Traumafolgen, Kristallopathien, rheumatoide Arthritiden, Spondyloarthritiden und Kollagenosen [7]. Die klinischen Zeichen mit der höchsten Sensitivität für eine bakterielle SA sind eine Schmerzexazerbation bei Bewegung, Rötungen und Schwellungen sowie ein Gelenkerguss. Die größte diagnostische Vorhersagekraft hat ein leukozytenreiches Gelenkpunktat. Laborparameter wie CRP-, Procalcitoninwert, periphere Leukozytenzahl und BSG haben aufgrund von niedriger Sensitivität und/oder Spezifität nur in der Zusammenschau der Befunde einen diagnostischen Nutzen [1]. Die Diagnose der bakteriellen SA beruht auf der mikrobiellen Erregerkultur aus dem Gelenk oder bei typischem klinischem Bild auf dem Erregernachweis in Blutkulturen, wenn kein Keimnachweis im Gelenkpunktat zu erlangen ist [8]. Die Infektion ist oft monomikrobiell. *Staphylococcus aureus* ist der häufigste auslösende Erreger (je nach Literaturangabe, Ursache der SA und betroffenem Gelenk bis 95 %). *P. aeruginosa* ist ein gramnegativer Nonfermenter und ein seltener Auslöser der bakteriellen SA (3–4 %), der meist nosokomial, oft im Zusammenhang mit invasiven Maßnahmen erworben wird [5, 10, 11]. Einzelne Fälle von rezidivierenden SA mit *Pseudomonas aeruginosa* sind beschrieben [12]. Die Therapie der SA beruht auf dem arthroskopischen oder offen chirurgischen Débridement mit Synovialektomie des betroffenen Gelenks sowie einer resistenzgerechten systemischen Antibiotikatherapie.

## Fazit für die Praxis

- Bei unklarer Monoarthritis, bakterieller septischer Arthritis in der Vorgeschichte und prädisponierenden Faktoren für eine hämatogene Streuung muss ein Rezidiv ausgeschlossen werden.
- Die frühzeitige Diagnose der SA ist prognoseentscheidend. Ein Keimnachweis sollte unbedingt durch Punktion oder ggf. chirurgisch angestrebt werden.
- Die Mortalität und Morbidität der SA ist unbehandelt hoch.
- Die Therapie erfolgt chirurgisch und antimikrobiell nach Resistogramm.
- Portsysteme können durch Biofilme besiedelt werden. Bei Nachweis einer bakteriellen SA sollte eine Explantation erwogen werden, um Rezidive zu verhindern.
